# Medical libraries and their complicated past: an exploration of the historical connections between medical collections and racial science

**DOI:** 10.5195/jmla.2023.1728

**Published:** 2023-07-10

**Authors:** Raymond Pun, Patrice R. Green, Nicollette Davis

**Affiliations:** 1 rpun@aldergse.edu, Alder Graduate School of Education, Redwood City, CA.; 2 patricergreen5@gmail.com, African American and African Diasporic Collections, Harvard Radcliffe Institute, Cambridge, MA.; 3 ndavis10@lsu.edu, Assistant Librarian, Louisiana State University, Baton Rouge, LA.

**Keywords:** Library history, medical libraries, racial science, medical collections, racism

## Abstract

For over a millennium, libraries and library workers have advanced the knowledge of human science by building, preserving, and sharing collections and research. Historically, libraries have also aligned their institutional responsibilities to adhere to and support the values and virtues of oppressive and colonial practices. Library history has shown the mistreatments and denials of information access of marginalized groups. The history of libraries in the health and medical sciences reveals how these institutions and their workers have preserved and circulated research studies perpetuating racial science. This commentary highlights how such institutions shape and contribute to racial science in the field of medicine. By exploring the history of medicine through this lens, we examine how such institutions have been complicit in upholding racial science. We explore historical documents and archival collections that have been collected and preserved, particularly records and data of vulnerable groups, to advance the knowledge and understanding of the human body through the ideology of racial science. We argue that health and medical sciences librarians need to critically interrogate the racism in medical libraries and its history and address how health misinformation is common even in scholarly publications.

## INTRODUCTION

Revisiting an institution's past is complicated. When we see institutions such as libraries playing a role in upholding racism in history, how do we make sense of their actions and consequences? Certainly, we acknowledge these past actions, but past actions continue to have an impact on present society. From the restrictions of access to libraries to demeaning library classification schemes, people of color in the United States, particularly Black people, have been (and still are) subjected to mistreatment and institutionalized racism [1]. Libraries have long been perceived as a symbol of democracy [2]. However, we must recognize that no institution and its history are perfect, and library workers' past actions have lingering consequences. To better understand how these institutions operated in society, along with the types of values they may have espoused, it is critical to re-examine the history of libraries and their role in de-centering racialized human experiences, particularly in the medical context.

The history of medical libraries can be traced to its earliest establishments from 2000 B.C., when medical writings were uncovered in an “ancient library of King Assurbanipal of Assyria (668-626 B.C.) in the ancient city of Nineveh” [3]. This library preserved a wealth of historic medical information dating back generations, like many libraries that were developed in the medieval and modern periods. However, in preserving history, libraries have supported ideas that were widely accepted at the time but problematic through a contemporary lens, especially recognizing past information behaviors that were guided by assumptions and values that need to be questioned [4]. Critical analyses and discussions on the libraries' contribution to upholding white supremacy and racial science values are apparent based on emerging literatures, especially in the history of anti-Blackness in libraries [5].

In colonial America, profiting from enslaved Africans as well as the practice of slavery as a method to physically build libraries are closely examined in *Slavery and the Making of Early American Libraries* by Sean D. Moore [6]. Wealth generated through slavery created an economic boom to pay for the expenses of constructing libraries and building their collections. Moore explains “the libraries thus served an important social, cultural, and civic function, creating a network of leaders who would become patriots or loyalists in the Revolution, providing the reading matter that would constitute them intellectually and ideologically, and planting the seeds for discourse on the slavery that facilitated early American cultural capitalization” [7]. Even before the development of libraries in colonial America, funds from slave trading supported and built libraries and their collections in imperialist countries.

Moreover, knowledge building in medicine and medical libraries took a similar turn. The development of collections in libraries perpetuated systemic racism in the medical field at large. Through colonial practices and experiences, scientists created biological classifications based on race and fostered a racial hierarchy scheme for research purposes.

Racial science is a pseudoscientific belief that defines and categorizes people in a hierarchy based on race. Widely embraced by colonialists, eugenicists, and white supremacists, racial science was viewed and known as “science” in the seventeenth century [8]. Racial inferiority and racial superiority were embedded as standard practices in academia, including the field of medicine [9]. We argue that medical libraries are complicit in upholding race science knowledge by shaping and contributing to inaccurate scholarship and practice in the medical and health sciences fields. Libraries' roles in history must be critiqued for their problematic past in supporting unethical practices through their collections of texts, knowledge building, sharing, and preserving degenerative analyses of vulnerable groups. Select sources highlight how libraries have engaged in these activities that support racial science or scientific racism. Racial science became a foundation for scientific approaches with troubling policy implications, social ramifications, and consequences among the communities and users, especially vulnerable populations, that libraries serve. To understand medical libraries' roles in upholding racial science values, we examine how collection-building created opportunities to preserve inaccurate knowledge and provide suggestions for libraries to interrogate health misinformation that persist today.

## RACIAL SCIENCE AND MEDICAL LIBRARIES

Racial science in libraries and other information institutions can be traced in both the Americas and Europe. Encrypted beneath the veil of empirical studies and data, racial science was widely adopted, accepted, and applied in library activities such as cataloging practices and collection development.

From a racial science lens, using human bodies without consent to enhance medical knowledge was standard in nineteenth-century United States. Medical students acted as grave robbers to raid graves and use corpses as dissection materials in medical schools. These bodies, treated as cadavers, were often Black people and bodies from the South were generally shipped to the medical schools in the North after the Civil War [10]. Furthermore, there was a general belief that Black bodies were “biologically inferior” by the end of the antebellum period [11]. The experimentations of human bodies would be part of the learning process to advance the medical field where they were used to build new knowledge and share such knowledge in textbooks that would eventually be collected, processed, and circulated by libraries without the consent of such cadavers.

Experiments with Black people and their bodies in the United States had occurred in other cases such as the Tuskegee Study of Untreated Syphilis in 1932-1972 and Henrietta Lacks' immortal cell line known as HeLa in 1951. In the Tuskegee Study, U.S. Public Health Service conducted a study on the effects of untreated syphilis on more than 400 Black men in Macon County, Alabama, and around Tuskegee. They were not aware of such tests and were left untreated [12]. Henrietta Lacks, a Black tobacco farmer from Southern Virginia, was diagnosed with cervical cancer and a doctor from Johns Hopkins “took a piece of her tumor without telling her [nor her family gave consent]” [13]. Both cases experimented with Black people to advance medical research yet the victims were exposed and/or did not give consent to be exploited. As a result, medical ethics have been incorporated in subsequent training required by Institutional Review Boards (IRB) for all researchers studying human subjects.

Cataloging guidelines also reinforced racial science through physical attributions and contributed degenerative analysis for research. In 1901, the *List of Subject Headings for Use in Dictionary Catalogs Prepared by a Committee of the American Library Association* showed “color of man” as a subject heading. Under that heading, other subordinate terms were listed: “referred from complexion; ethnology; face; man; negroes; physiology; skin” [14]. The biological markers are included under this term and problematizes how librarians, particularly catalogers, classified and processed books and materials about Black people, and how they were identified through physical qualifiers. According to historian Harriet Washington, the depiction of physical qualifiers such as hair, facial angles, stature, and stance of enslaved Black people were often compared to animals. These depictions were used to disseminate and reinforce the evolutionary and biological connections after the World's Fair in Milan in 1906. Washington writes, “such uncomplimentary images were published in scientific journals and would soon adorn children's textbooks” [15]. Textbooks, whether they are for scientists or school children, can reinforce systemic racism in formal learning environments. Libraries and their activities in collecting such scientific journals and textbooks played a role in perpetuating racial science in America.

Similar agendas were found in Europe under Nazi Germany where the Nazi scientists learned about and adopted this heinous thinking from the United States in their own policies [16]. Known for their racial superiority ideology in promoting the Aryan race, medical textbooks created by the Nazis, such as Eduard Pernkopf's *Topographische Anatomie des Mensche* (*Topographic Anatomy of Man*) in 1933, contained vivid examples of anatomical drawings that were based on the bodies of executed prisoners by the Nazis. The bodies were often from Jewish people and echoed the same unethical issue of using bodies without consent to educate medical students. Pernkopf, a physician and dean of the medical faculty of the University of Vienna, “summarized the role of medicine in the new state as being both positive and negative, that is, both ‘furthering the propagation of the fit' and ‘eliminating the unfit and defective' by controlling marriage, by forbidding ‘breeding by individuals who do not belong together properly,' and by sterilizing the genetically inferior” [17]. Astonishingly, some physicians still consult this book because of its detailed diagrams today [18].

## THE ROLE OF JOURNAL COLLECTIONS PERPETUATING MEDICAL RACISM

Medical libraries and archives, including those from medical societies, subscribed and managed journal collections for their research community of users. Journals that were published in the American South promoted specific scientific racism and revealed physicians' perspectives and practices in perpetuating medical racism. *Transylvania Medical Journal* (1818–1862), *Charleston Medical Journal and Review* (1848-1877), and *The New Orleans Medical and Surgical Journal* (1844-1973) are examples of journals that were collected, disseminated, and studied, particularly by white southerners, prior to the American Civil War [19]. Universities such as Louisville Medical Institute (1837-1846) and now the Department of Medicine at the University of Louisville collected *Transylvania Medical Journal* [20].

On March 19, 1851, *The Mississippi Free Trader and Natchez Gazette* described the *Charleston Medical Journal and Review* as a “work…that forcibly recommends itself to all interested in the progress of science and medicine”[21]. *The New Orleans Medical and Surgical Journal* also received a glowing review for its July issue by *The Mississippi Free Trader and Natchez Gazette* on July 17, 1847, wherein the scholarship “fully maintains its character as an ably conducted and valuable work – worthy of the liberal patronage of the profession”[22]. These journals published and disseminated works that purported ideas about the health of enslaved Black people. Such publications reinforced racist ideology and hierarchy within the medical literature. For example, the first issue of *Transylvania Medical Journal* published Samuel George Moton's speech in 1849, which covered the brain size of individuals in the *Proceedings of the Academy of Natural Sciences* in October 1849:

No means has been taken of the Caucasian race collectively because of the very great preponderance of Hindu, Egyptian and Fellah skulls over those of the Germanic, Pelagic and Celtic families. Nor could any just collective comparison be instituted between the Caucasian and Negro groups in such a table unless the small-brained people of the latter division were proportionate in number to the Hindus [sp], Egyptians and Felluhs of the other group. Such a computation, were it predictable, would probably reduce the Caucasian average to about 87 cubic inches, and the Negro to 75 at most, perhaps even to 75, and thus confirmatively establish the difference of at least nine cubic inches between the mean of the two races [23].

Morton's problematic arguments echo racial difference and hierarchy through physical features. This type of thinking was prominently featured and accepted in such journals and became works to be collected and circulated, reinforcing systemic racism in the medical community [24]. *The New Orleans Medical and Surgical Journal* published articles by physicians such as Samuel Cartwright whose writings focused on racialized medical thoughts and perpetuated scientific racism [25]. This journal was used and perceived as a resource for physicians to read about racial disparities and differences between whites and enslaved Blacks. *Southern Medical and Surgical Journal* was the first journal that was published in the South that “served an exclusively slave population at the Jackson Street Hospital…” [26]. In essence, slave hospitals were created to protect and uphold the economic interests of slavery and provide knowledge-building opportunities for physicians to expand their medical skills [27]. This journal was published by Augusta's Medical College of Georgia in 1836 and the purpose of this serial was to “present scholarly work in the art of medicine to urban and rural medical practitioners” [28]. Augusta's Medical College established its library in 1834 and starting in 1835, medical students had full access to the library collection [29].

Other physicians who reinforced scientific racism include J. Marion Sims. Sims is a notable example who was largely viewed as an innovative surgeon in the medical field in the nineteenth century, particularly known for his *vesicovaginal fistulas* technique, a complicated procedure for those experiencing prolonged labor [30]. This medical research had major implications in surgery but a deeply unethical and troubling past. From 1845-1849, Sims tested this technique on 10 Black women who were enslaved. Only three were named in Sims' writings: Anarcha Westcott, Betsey Harris, and Lucy Zimmerman [31]. These experimental surgeries were performed on them without using anesthesia [32]. However, it was later known that Sims performed the technique on white women and did use anesthesia. The technique was also unsuccessful in the beginning. Sims eventually perfected his technique after 30 surgeries on one person, Anarcha Westcott [33]. Moreover, he wrote his findings to be shared in medical textbooks and journals such as the *American Journal of Medical Sciences* and *New York Medical Journal* [34]. Despite his cruel and painful experiments, he was recognized and honored for his medical breakthroughs and perceived as a champion for women's health and recognized as the “father of modern gynecology” in his *New York Times* obituary in 1883 [35].

In reality, the women suffered in pain, and their voices and ordeals were not captured by Sims; they were largely absent. The findings of this surgery in such journals would inevitably reward such physicians [36]. Most recently, historians and medical researchers are reexamining and questioning Sims' impact and legacy in the field by arguing how he violated medical ethics and perpetuated heinous and racist acts on Black women [37]. Sims may have contributed to the field in medicine, yet his research exploited and abused vulnerable groups, and at their expense as their bodies were used as experiments. Medical libraries collected and preserved such information to advance the knowledge of the human body yet perpetuated racial science and violence on marginalized groups.

## CALL TO ACTION: INTERROGATING MEDICAL LIBRARIES' PAST AND PRESENT

Libraries can revise their collection development policies to provide a clear statement that the texts in medical libraries and archives have reinforced racial science in health and medical fields. This would serve as a critical reminder to the profession of its painful and complicated past. Moreover, the profession at large must recognize the painful experiences of those who were experimented on, mistreated and suffered as a result of “advancing” science of the human condition. Recognizing and reckoning how the legacy of slavery has directly influenced and impacted Black people in the sciences and medical librarianship field today must be contended.

Recent publications such as *A History of Medical Libraries and Medical Librarianship from John Shaw Billings to the Digital Era* by Kronenfeld and Kronenfeld cover extensively the history of medical librarianship and its innovation but do not discuss the issues of medical racism in libraries. Future publications covering the history of medical libraries, particularly in the United States, may need to acknowledge how such medical collections over time perpetuated racial science in the field [44]. Although this history is not included in Meyerhoff's “Foundations of Medical Librarianship” published in 1977, future publications regarding the foundation of medical librarianship should mention the complicit role of medical libraries in upholding racial science.

In addition to acknowledging the racist ideas that plague library collections, it is necessary for librarians to teach about how this misinformation continues to manifest in health information. Racial science has been used as a foundation for health science information, it's important to think critically about how we teach health science, not only for us as librarians to think critically but also teach students to evaluate information critically. Harmful and false ideas about Black bodies continue to be a part of the medical world today. As librarians, how do we prepare students to be better practitioners and researchers? Whether it's a one shot instruction session or a research consultation, it can seem difficult to incorporate such a topic in a short timeframe.

In academic and health science library instruction, exposing students to the history of medical science research can help students to understand how and why health misinformation is common even in scholarly publications. Research over the past few decades have shown that there are racial disparities when it comes to treatment and pain management relating back to the misinformation from racial science that Black bodies have a higher pain tolerance than white bodies [38]. In 2016, a study showed that white physicians continue to undertreat Black patients due to this bias. This finding helps current and future practitioners to reflect on their approaches and hopefully influence impactful changes. Teaching students and faculty about the racist history of information can help them to bridge the gaps in knowledge and highlight how these ideologies are still imbedded in medical practice and research.

Building information searching and evaluation skills through a critical lens can potentially influence better practices for medical students. In 2018, librarian Dawn Stahura developed the ACT UP method as an acronym to evaluate sources. Stathura writes, “By definition, ACT UP means to act in a way that is different from “normal,” the normal established by patriarchy and the systemic oppression of marginalized groups. To ACT UP is to actively engage in dismantling the oppression of people of color and acting upward to create a more socially just system. […] using the acronym ACT UP provides an opportunity to tie evaluating sources with grassroots activism” [39]. Exposing students and even faculty to a wide variety of sources, specifically from historically marginalized people can introduce new and thought-provoking ideas. Organizations such as the Cite Black Women Collective encourage researchers to read works by Black women and center their knowledge and experiences. Founder Christen A. Smith writes:

What does it look like to dismantle the patriarchal, white supremacist, heterosexist, imperialist impetus of the neoliberal university (and its accomplices) by centering Black women's ideas and intellectual contributions? Historically, the university has exploited Black women's labor, appropriated our ideas and refused to give us the appropriate credit for our work. Cite Black Women is, therefore, a project of radical refusal with revolutionary possibilities. If universities and oppressive spaces of knowledge production seek to silence and erase Black women than acknowledging and centering us holds revolutionary possibilities as a radical praxis of Black feminist utopian imagining/marronage [40].

Incorporating real-world scenarios and examples related to how harmful misinformation is to the health science field can provide students with an opportunity to reflect on how their information-seeking skills can have lasting impacts. As previously mentioned, enslaved Black women were used to perfect surgical procedures related to labor and today Black women have the highest mortality rate during pregnancy and childbirth. In addition to factors related to healthcare access and income, race also plays a role in quality of care and treatment [41].

When we think about how to engage our students in the classroom and in our instruction sessions, it's crucial to unearth the voices and experiences of those who are often overlooked and undervalued. Similar to librarianship, the medical field continues to be a largely white profession where the status quo keeps diverse voices underrepresented in various capacities. While we are not able to change the past, vocalizing these injustices with transparency and a critical lens can serve as a means towards harm reduction in the health sciences.

## CONCLUDING THOUGHTS

How should medical ethics address harrowing practices from the past documented within the collections of medical libraries? This commentary shared select examples of problematic issues within the medical libraries' history. Recognizing and acknowledging the pain and suffering of individuals and groups at the hands of scientists and physicians is key. The Medical Library Association's (MLA) Code of Ethics for Health Sciences Librarianship promotes a framework that guides ethical decision making in the profession [42]. MLA may consider fostering the notion that medical libraries are not neutral and have played an instrumental role in perpetuating scientific racism and harm in the field.

At the 2018 Medical Library Association's Conference Janet Doe Lecture, Elaine Russo Martin describes, “the notion that the medical library is a social institution that serves as a community center for its users is not neutral. Medical librarians cannot be neutral and be trusted advocates for their communities, especially the underserved. We believe that medical librarians can be a force for social good. This is crucial to our future and to the health of our local communities and a sustainable global community” [43].

Discussing these historical matters will not be a simple checklist process but it is a necessary step. By ignoring the complicity of medical libraries in aiding and supporting medical research that reinforced racial science in the field historically, the profession reinforces racial injustice and inequity, and ultimately, maintains structural racism in the profession and in public health at large. Critically studying and examining any library's history and its past practices offers deeper analyses and opportunities to reflect on those who were affected, what to acknowledge, and how to reconcile these recurring injustices with deep ramifications for today. All institutions are subject to critique and analysis, and libraries and their collections are not exempt from these discussions.
